# Robot‐Assisted Radical Nephrectomy for Renal Cell Carcinoma in a Right Intrathoracic Kidney Following Congenital Diaphragmatic Hernia Repair: A Case Report

**DOI:** 10.1002/iju5.70081

**Published:** 2025-08-06

**Authors:** Yutaro Sasaki, Saki Kobayashi, Fumiya Kadoriku, Kei Daizumoto, Ryotaro Tomida, Yoshito Kusuhara, Tomoya Fukawa, Kunihisa Yamaguchi, Yasuyo Yamamoto, Junya Furukawa

**Affiliations:** ^1^ Department of Urology Tokushima University Graduate School of Biomedical Sciences Tokushima Japan

**Keywords:** congenital diaphragmatic hernia, intercostal trocar placement, intrathoracic kidney, minimally invasive surgery, robot‐assisted radical nephrectomy

## Abstract

**Introduction:**

Congenital diaphragmatic hernia (CDH) can result in intrathoracic displacement of the kidney, presenting anatomical challenges for robot‐assisted radical nephrectomy (RARN). Reports of RARN in such cases are scarce.

**Case Presentation:**

A 56‐year‐old man with a history of right‐sided CDH repair was referred for evaluation of an incidentally discovered right renal mass. Computed tomography revealed two right renal tumors (cT1aN0M0) and cranial displacement of the kidney into the thoracic cavity. Given the possibility of intra‐abdominal adhesions and the retrohepatic location of the kidney, retroperitoneal RARN was selected. An intercostal trocar was used to access the high‐positioned kidney. The renal vessels were safely managed using a Vas Guide, and no complications occurred. Pathology confirmed clear cell renal cell carcinoma (pT1aN0M0).

**Conclusion:**

RARN can be safely performed in patients with prior CDH repair and intrathoracic renal displacement. Preoperative planning and alternative trocar strategies, such as intercostal placement, are essential for successful outcomes.

**Abbreviations**
CDH: congenital diaphragmatic hernia
RARN: robot‐assisted radical nephrectomy

**Summary**

- Robot‐assisted radical nephrectomy can be safely performed for renal cell carcinoma in a right intrathoracic kidney following congenital diaphragmatic hernia repair.
- Given the complex anatomy, tailored surgical planning—including retroperitoneal and intercostal access—is essential.

## 1 Introduction

In patients with a history of congenital diaphragmatic hernia (CDH) repair, the kidney may be displaced cranially into the thoracic cavity due to diaphragmatic and thoracoabdominal anatomical changes [1, 2, 3]. This displacement can complicate standard trocar placement and access routes during robot‐assisted radical nephrectomy (RARN). To date, reports of RARN in patients with such complex anatomy remain limited. We herein present a case of successful retroperitoneal RARN for right renal cell carcinoma in a patient with a right intrathoracic kidney following CDH repair, highlighting the surgical approach and perioperative considerations.

## 2 Case Presentation

**FIGURE 1:**
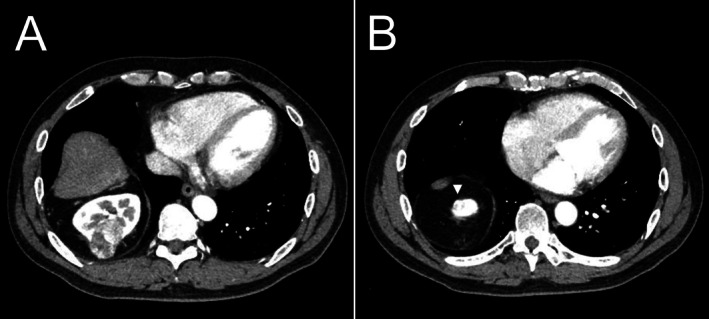
Contrast‐enhanced computed tomography showing two right renal tumors consistent with renal cell carcinoma. (A) A 39‐mm tumor with a R.E.N.A.L. nephrometry score of 8p. (B) A 15‐mm tumor with a R.E.N.A.L. nephrometry score of 4a (indicated by an arrow).

A 56‐year‐old Japanese man was referred to our institution following incidental detection of a right renal mass during a routine health checkup. Contrast‐enhanced computed tomography revealed two right renal tumors, both clinically staged as cT1aN0M0 and consistent with renal cell carcinoma: a 39‐mm tumor with a R.E.N.A.L. nephrometry score of 8p (Figure 1A) and a 15‐mm tumor with a score of 4a (Figure 1B).

**FIGURE 2:**
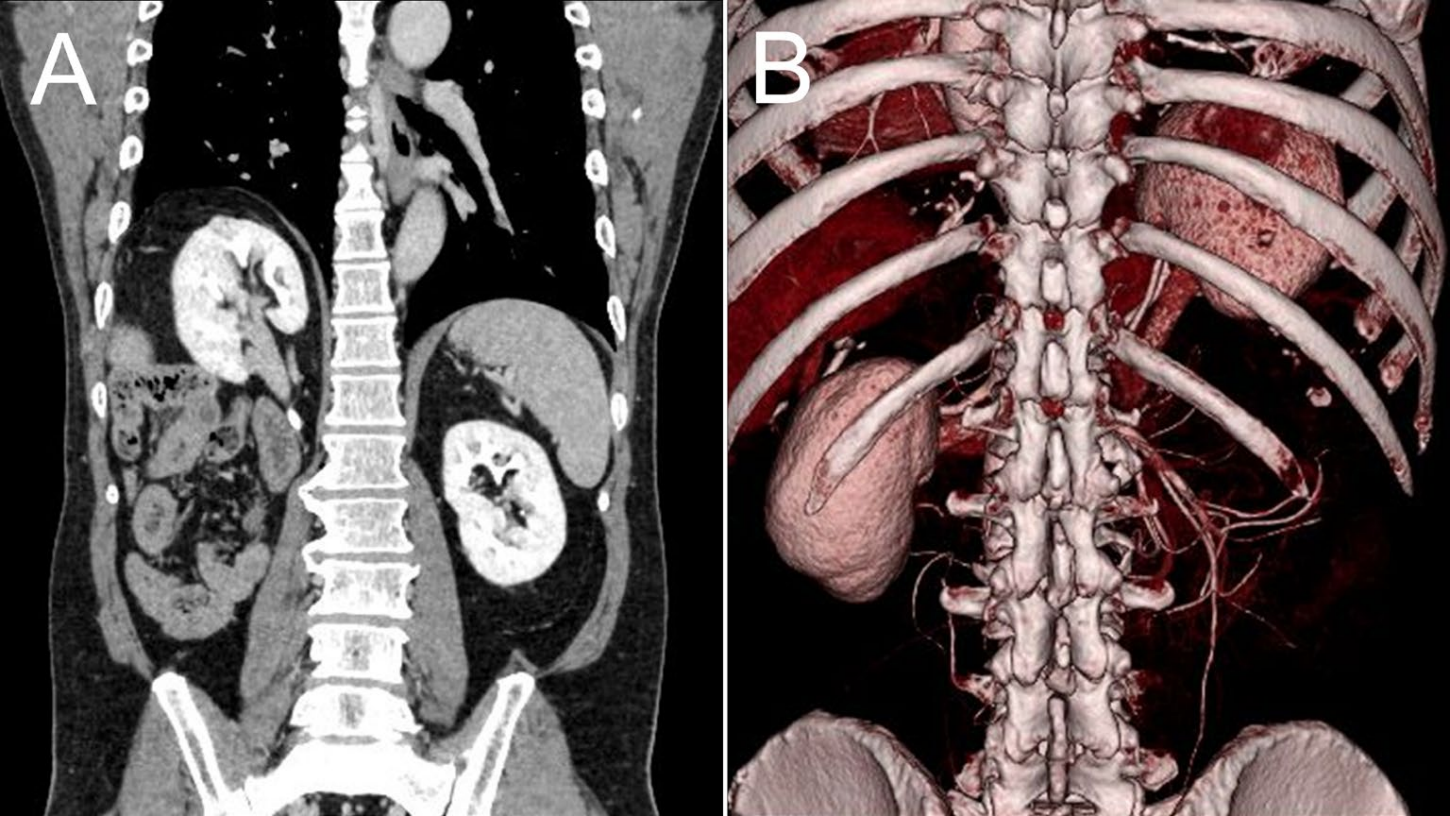
Imaging demonstrating cranial displacement of the right kidney. (A) Coronal computed tomography view showing the right kidney displaced into the thoracic cavity, reaching the level of the heart. (B) Three‐dimensional reconstructed computed tomography image clearly demonstrating the intrathoracic location of the right kidney.

The patient had a history of right‐sided CDH, which had been repaired via open abdominal surgery on the second day of life. Because of the considerable time that had elapsed since the procedure, further operative details were unavailable. His height, weight, and body mass index were 166.5 cm, 77.4 kg, and 27.9 kg/m^2^, respectively. A 41‐cm transverse surgical scar was noted below the costal margin. Notably, the right kidney was markedly displaced cranially, reaching the level of the heart—presumably as a result of the prior hernia repair (Figure 2A). A three‐dimensional reconstructed computed tomography image clearly demonstrated that the kidney was located within the thoracic cavity (Figure 2B). After thorough discussion with the patient, a decision was made to prioritize oncological curability. Therefore, RARN was chosen over robot‐assisted partial nephrectomy.

**FIGURE 3:**
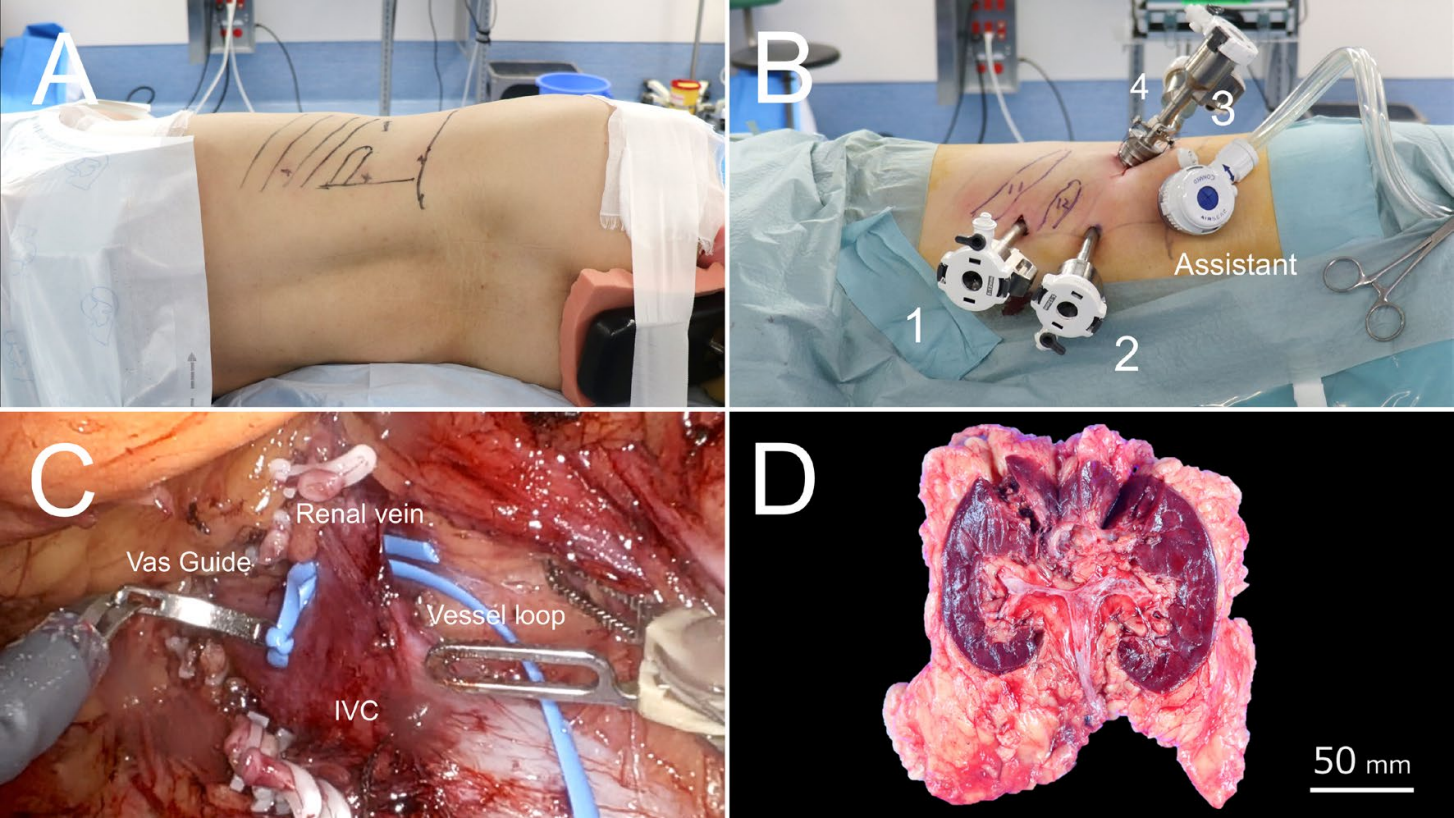
Key intraoperative findings and gross specimen. (A) Patient positioning for retroperitoneal robot‐assisted radical nephrectomy: left lateral decubitus in the jackknife position. (B) Trocar placement, including intercostal insertion through the 11th intercostal space. (C) Intraoperative view showing the right renal vein looped with vessel loops using a Vas Guide. (D) Gross specimen of the resected right kidney. Final pathology confirmed two tumors of clear cell renal cell carcinoma, both staged as pT1a, with Fuhrman nuclear grade 3 and negative surgical margins.

RARN was performed via a retroperitoneal approach with the patient in the left lateral decubitus jackknife position (Figure 3A). Anticipating limited cranial access to the kidney with standard trocar placement, the initial trocar was inserted through the 11th intercostal space (Figure 3B). Given the risk of pleural or pulmonary injury associated with trocar insertion near the diaphragm, preparations for single‐lung ventilation were made preoperatively.

Following retroperitoneal entry via an open technique, a balloon dissector was used to create the working space. Two renal arteries and one renal vein were identified and looped using vessel loops with the aid of a Vas Guide (Figure 3C); then ligated with Hem‐o‐lok clips (Teleflex, Morrisville, NC, USA).

During kidney dissection, the ascending colon, duodenum, and liver were carefully retracted to prevent injury. The adrenal gland was preserved. Cranial dissection of the kidney was completed through the intercostally placed trocar without the need for additional trocars. The specimen was retrieved in an endoscopic bag (Inzii Retrieval System; Applied Medical Inc., Rancho Santa Margarita, CA, USA) and extracted via a 6.0‐cm extension of the third trocar site.

The total operative time was 227 min, with a console time of 123 min. The estimated blood loss was 10 mL. The postoperative course was uneventful, and the patient was discharged on postoperative day 12. The gross specimen revealed two distinct tumors in the resected right kidney (Figure 3D). Final pathology confirmed both as clear cell renal cell carcinoma, staged as pT1a, with Fuhrman nuclear grade 3 and negative surgical margins.

## 3 Discussion

CDH is a developmental defect of the diaphragm that allows abdominal organs to herniate into the thoracic cavity, often resulting in pulmonary hypoplasia and displacement of intra‐abdominal organs [1, 2, 3]. In rare cases, the kidney may also be displaced into the thoracic cavity—a condition known as an intrathoracic kidney [1, 2, 3]. This anatomical anomaly can complicate surgical procedures because of the atypical location and altered relationships with surrounding structures.

In this case, the patient had undergone right‐sided CDH repair during the neonatal period, resulting in cranial displacement of the right kidney to a level near the heart. This posed significant challenges for surgical management of the renal tumor, necessitating meticulous preoperative planning and modification of standard techniques.

To our knowledge, there are no prior reports of RARN in a patient with a history of CDH repair. Several factors influenced our decision‐making regarding the surgical approach. The patient had experienced five episodes of ileus following CDH surgery, all managed conservatively, suggesting the presence of significant bowel adhesions. Additionally, the right kidney was located posterior to the liver, which would have necessitated hepatic mobilization if a transperitoneal approach had been employed. Although a transperitoneal approach could potentially offer a wider working space, to avoid these issues, a retroperitoneal approach was chosen. However, given the high position of the kidney, standard trocar placement was expected to be inadequate, leading to the selection of intercostal trocar placement instead.

Recognizing the risk of pleural or pulmonary injury associated with intercostal trocar placement, single‐lung ventilation was prepared for but ultimately not required because the trocars were successfully inserted without injury. During the procedure, special care was taken to avoid collisions between the robotic arms and the ribs or iliac crest because the kidney's cranial position constrained maneuverability. In this case, the procedure was successfully completed using the 1st (intracostal, left arm), 2nd (camera), 3rd (right arm), and 4th (extra‐arm) trocars. To improve operability under the constrained anatomical conditions, alternative strategies—such as switching the 3rd and 4th arms or employing the port‐in‐port technique to convert an assistant port into the 3rd arm—may also be considered as viable options. The limited working space also led to frequent instrument interference, particularly with the assistant's forceps. To loop the renal vessels safely and efficiently under these conditions, the Vas Guide proved highly effective [4].

Thoracic approaches for partial nephrectomy in intrathoracic kidneys have been reported [5, 6]. Sharma et al. [5] reported an open thoracic approach, while Sanz del Pozo et al. [6] described a thoracoscopic approach. However, no reports of robot‐assisted surgery have been published to date. As we had no prior experience with thoracic approaches, these options were not considered.

This case demonstrates that RARN is feasible even in patients with complex anatomical alterations resulting from prior CDH repair. Preoperative imaging and careful surgical planning were essential in tailoring the approach and minimizing intraoperative risks. With appropriate adaptation, minimally invasive surgery can be performed safely in such challenging cases.

## 4 Conclusion

RARN is a feasible and safe option for patients with a history of CDH repair, despite associated anatomical challenges. Careful preoperative planning and tailored surgical strategies are essential to ensure a successful outcome.

## Ethics Statement

The Ethics Committee of Tokushima University Hospital approved the study protocol (approval no. 4461). This research was conducted in accordance with the provisions of the Declaration of Helsinki.

## Consent

We obtained written informed consent from the patient for publication of this case report.

## Conflicts of Interest

The authors declare no conflicts of interest.
